# Key indicators contributing to prolonged emergency department stays in Saudi Arabia: a modified Delphi study

**DOI:** 10.1186/s12873-025-01438-y

**Published:** 2025-12-15

**Authors:** Lujain Aljohani, Nuwayyir Abdullah Alqasimi, Waleed Alharbi, Mohammed Alshalhoub, Abdulrahman Alrajhi, Nasser Alkahtani, Reem Alshehri, Ahmed Hussein Alkohlani, Majid Alsalamah

**Affiliations:** 1 College of Medicine, Princess Noura bint Abdulrahman University, Riyadh, Saudi Arabia; 2 College of Medicine, King Saud bin Abdulaziz University for Health Sciences, Riyadh, Saudi Arabia; 3 Emergency Medicine Department, Ministry of the National Guard - Health Affairs, Riyadh, Saudi Arabia; 4 King Abdullah International Medical Research Centre, Riyadh, Saudi Arabia

**Keywords:** Emergency department, Length of stay, Prolonged LoS, Indicators, Saudi Arabia

## Abstract

**Background:**

A prolonged length of stay (LoS) in emergency departments (EDs) is a growing issue that affects patient prognosis, resource allocation, and operational efficiency. In Saudi Arabia, an ED stay exceeding 8 h is considered prolonged; however, few studies have identified the key elements contributing to this issue. This study aimed to determine and rank indicators associated with prolonged LoS in EDs based on the consensus of emergency medical practitioners in Riyadh, Saudi Arabia.

**Methods:**

A two-round modified Delphi study was conducted from September 2024 to January 2025. Purposeful sampling included emergency physicians and nurses (129 in Round 1 and 137 in Round 2). Participants evaluated 34 potential indicators of prolonged ED stays using a Likert-scale questionnaire. Consensus was defined a priori, and the top 10 indicators were reassessed in Round 2. Statistical analysis using SPSS included medians and interquartile ranges (IQR), with significance set at *p* < 0.05.

**Results:**

A total of 129 and 137 participants completed Rounds 1 and 2, respectively. The most important indicator identified was the percentage of the ED occupied by inpatients. Other highly ranked indicators included the total ED patient count, average time in the ED for admitted patients, and the number of boarding patients. These indicators consistently showed high consensus, with a median (IQR) of 5 (1). Female ED workers rated inpatient occupancy, time between decision to disposition, and boarding patients as higher contributors to prolonged LoS (*p* < 0.03). ED nurses rated the average time in the ED for admitted patients, consult-to-disposition time, and physician availability as stronger indicators of prolonged LoS in the ED (*p* < 0.014).

**Conclusion:**

Inpatient boarding, reflected by the percentage of the ED occupied by inpatients and the number of boarding patients, was the primary factor contributing to prolonged LoS in Saudi EDs. These findings emphasize that prolonged LoS in the ED is a hospital-wide challenge requiring coordinated action from healthcare administrators and policymakers to expedite inpatient discharges and optimize ED processes. Enhancing national programs such as Ada’a by incorporating these indicators into performance metrics may improve ED efficiency and patient care in Saudi Arabia.

**Supplementary Information:**

The online version contains supplementary material available at 10.1186/s12873-025-01438-y.

## Background

Emergency departments (EDs) are the frontline of healthcare systems and operate in high-pressure environments. EDs provide care for patients with life-threatening or urgent conditions and play a critical role in initiating timely treatment [1]. However, several challenges affect ED performance, including an increased length of stay (LoS) [2].

LoS is a key metric for optimizing patient outcomes, reducing care costs, and improving resource allocation. It measures the time from patient arrival to discharge [3]. Globally, prolonged ED stays are commonly defined as exceeding 6–8 h [4], whereas in Saudi Arabia, an ED stay of more than 8 h is considered prolonged. Extended LoS has been linked to delays in care, increased morbidity and mortality, higher healthcare costs, overcrowding, and staff burnout [5–7]. 

Multiple factors influence LoS, including patient-specific, clinical, hospital-related, and administrative variables [8]. Patient-related contributors include age, severity of condition, and triage level, while hospital-related factors include facility size and consultation patterns. Internationally, approximately 38.4% of ED patients experience prolonged LoS, and in Saudi Arabia, nearly 30% of patients spend four or more hours in EDs despite recent improvements in care quality [3]. This persistent challenge highlights the need to identify operational inefficiencies that continue to affect ED throughput.

Only a limited number of studies have comprehensively identified actors influencing LoS in Saudi Arabia [9, 10]. Therefore, this study aims to evaluate the level of consensus among ED personnel in Riyadh regarding the relative importance of potential indicators contributing to prolonged ED LoS. Identifying these indicators is essential to understanding the systemic obstacles affecting patient flow and to informing evidence-based strategies that can help healthcare facilities reduce prolonged stays and improve ED efficiency.

## Methods

### Study design and setting

A two-round modified Delphi study was conducted from September 2024 to January 2025. It aimed to assess factors associated with prolonged ED length of stay (LoS) by ranking indicators according to expert consensus. This method was selected to gather expert opinions, prevent peer pressure, and avoid influence by ensuring anonymity through an iterative feedback process. Responses were collected in rounds, and each round provided feedback to refine and align expert perspectives. The study was reviewed and approved by King Abdullah International Medical Research Center (KAIMRC) (Approval No: 0000047224).

A modified Delphi design was utilized in this study. In Round 1, a structured, literature-based questionnaire was used instead of open-ended item generation. After each round, participants received iterative feedback and anonymized summary statistics, with all communication conducted electronically to maintain anonymity and avoid face-to-face discussion. These adjustments improved efficiency while ensuring that participants’ identities remained confidential.

Participants were selected through purposive sampling, with 129 participants in the first round and 137 in the second, all of whom were physicians and nurses working in the ED. Their expertise and clinical observations were used to assess indicators of prolonged ED stays.

In this study, we followed methodological standards to determine the sample size rather than relying solely on statistical calculations. Established guidelines for Delphi studies recommend expert panels of 15–60 participants. Our study exceeded these recommendations, with 129 experts in Round 1 and 137 in Round 2. This larger sample strengthened the validity and reliability of the consensus process. A total of 129 participants in Round 1 and 137 participants in Round 2 were included, exceeding the commonly accepted range for Delphi panels, thereby enhancing the credibility of the findings. Each participant received a consent form outlining the Delphi study process and expectations for their involvement, along with contact details.

From the previous literature, a list of measures was generated as potential indicators of prolonged ED stays. Of the 41 items identified, 34 were agreed upon by experts. For each of the 34 potentially relevant variables, operational definitions were included in the survey questionnaire.

In previous literature, forty-one potential indicators of prolonged ED stays were identified. An independent expert panel reviewed these indicators to assess their relevance and potential overlap within the context of Saudi EDs. Through consensus, seven indicators were excluded because they were either redundant or had limited applicability. Therefore, thirty-four indicators were retained for further evaluation using the Delphi method. Operational definitions for each indicator were provided to participants in the survey questionnaire to ensure consistent interpretation. These definitions are presented in Supplementary File 1.

### First round

In the first round, participants rated the 34 measures on a Likert-type scale (5 = Strongly Agree, 1 = Strongly Disagree). Consensus was defined a priori as a median score ≥ 4, interquartile range (IQR) ≤ 1, and/or ≥ 80% agreement (“Agree” or “Strongly Agree”). Using these criteria, 10 indicators reached consensus and were advanced to Round 2.

### Second round

An anonymized summary was provided to participants in Round 2, including median values and agreement rates for each of the 10 indicators, along with feedback from Round 1. The second-round questionnaire was emailed to all eligible participants, including those who had not participated in Round 1. Non-respondents were issued up to three reminders at two-week intervals after the initial distribution.

Invitations for Round 2 were sent to all eligible ED staff, including those who did not participate in Round 1, to ensure broader representation. Up to three email reminders were sent at two-week intervals to reduce the likelihood of non-response. A demographic comparison between the two rounds including age, gender, and position showed minimal non-response bias, indicating that differences between respondents and non-respondents were small.

### Statistical analysis

Data were extracted and managed in Microsoft Excel. Statistical analysis was performed using SPSS (Statistical Package for the Social Sciences) version 26.0 (Armonk, NY, USA). Descriptive statistics were used to summarize participant characteristics and consensus ratings. Categorical variables were presented as frequencies and percentages, while continuous variables, including Likert-scale responses, were reported as medians and interquartile ranges (IQRs) due to their ordinal nature.

The primary outcomes analyzed were the consensus ratings for each prolonged ED LoS indicator. The Kruskal–Wallis test, a non-parametric method appropriate for ordinal data, was selected to assess differences in ratings between professional groups (physicians vs. nurses) and other demographic factors. A significance level of *p* < 0.05 was used for all statistical tests.

## Results

### First Delphi round

The first Delphi round included 129 ED workers. Participants evaluated 34 prolonged LoS indicators in EDs. The majority of participants were male (58.9%) and ER physicians (59.7%), with a median (IQR) age of 28 (8), as shown in Table 1.

**Table 1:** Presents the participants’ characteristics for round 1

Variables		Count	Percent
Age	Mean (SD)	30.81 (7.724)	
	Median (IQR)	28.00 (8)	
Gender	Male	76	58.9
	Female	53	41.1
Position	ER Physicians	77	59.7
	ER Nurse	52	40.3

**Fig. 1:**
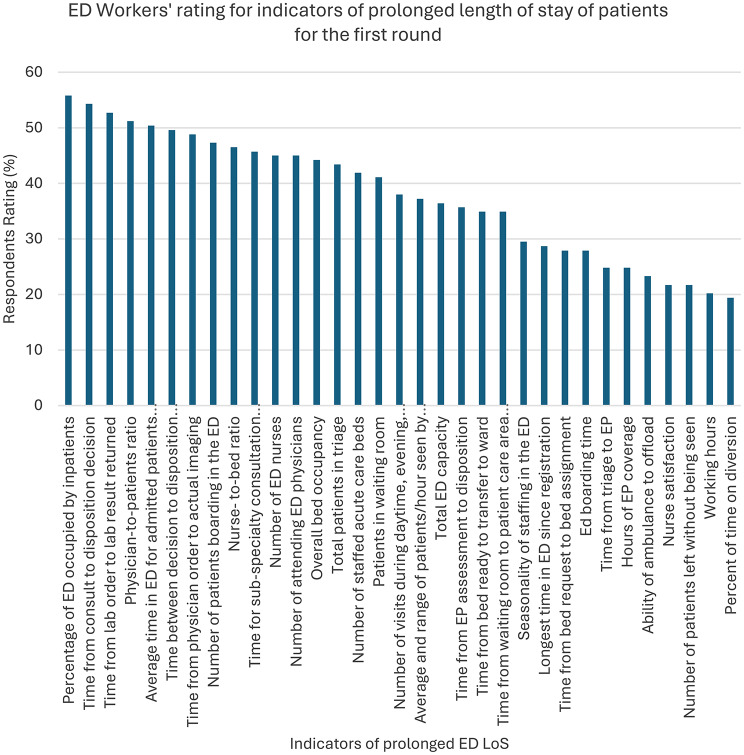
Illustrates ED Workers’ ratings for the indicators of prolonged LoS of patients in the ED for the first round

As shown in Table 2, the percentages and overall scores for each ED prolonged LoS indicator are listed. The total number of ED patients (55.8%), the percentage of the ED occupied by inpatients (55.8%), and the time from consultation to disposition decision (54.3%) had the highest consensus among the 34 indicators. In contrast, the percentage of time spent on diversion (19.4%), nurse satisfaction (21.7%), and the number of patients who left without being seen (21.7%) received the least consensus among all indicators (Fig. 1).

**Table 2:** Demonstrates the evaluation of ED prolonged LoS indicators for the first round

Which elements do you think are most associated with an increase in the length of stay in the emergency department?	Strongly Agree (5)	Agree (4)	Neutral (3)	Disagree (2)	Strongly disagree (1)	Overall score Median (IQR)
1. Total number of ED patients	72 (55.8)	37 (28.7)	12 (9.3)	6 (4.7)	2 (1.6)	5 (1)
2. Overall bed occupancy	57 (44.2)	44 (34.1)	15 (11.6)	10 (7.8)	3 (2.3)	4 (1)
3. Number of patients boarding in the ED	61 (47.3)	47 (36.4)	12 (9.3)	7 (5.4)	2 (1.6)	4 (1)
4. Percentage of ED occupied by inpatients	72 (55.8)	36 (27.9)	15 (11.6)	4 (3.1)	2 (1.6)	5 (1)
5. Time from bed request to bed assignment	36 (27.9)	39 (30.2)	35 (27.1)	15 (11.6)	4 (3.1)	4 (2)
6. Number of staffed acute care beds	54 (41.9)	40 (31.0)	18 (14.0)	16 (12.4)	1 (0.8)	4 (2)
7. Time from triage to EP	32 (24.8)	18 (14.0)	43 (33.3)	27 (20.9)	9 (7.0)	3 (3)
8. Time from bed ready to transfer to ward	45 (34.9)	40 (31.0)	31 (24.0)	10 (7.8)	3 (2.3)	4 (2)
9. Nurse satisfaction	28 (21.7)	38 (29.5)	44 (34.1)	17 (13.2)	2 (1.6)	4 (1)
10. Time from waiting room to patient care area in ED	45 (34.9)	40 (31.0)	28 (21.7)	12 (9.3)	4 (3.1)	4 (2)
11. Patients in waiting room	53 (41.1)	40 (31.0)	23 (17.8)	11 (8.5)	2 (1.6)	4 (2)
12. Average time in ED for admitted patients since admission	65 (50.4)	37 (28.7)	19 (14.7)	6 (4.7)	2 (1.6)	5 (1)
13. Total patients in triage	56 (43.4)	38 (29.5)	21 (16.3)	12 (9.3)	2 (1.6)	4 (2)
14. Number of patients left without being seen	28 (21.7)	28 (21.7)	30 (23.3)	28 (21.7)	15 (11.6)	3 (2)
15. Time from consult to disposition decision	70 (54.3)	33 (25.6)	12 (9.3)	13 (10.1)	1 (0.8)	5 (1)
16. Average and range of patients/hour seen by EP	48 (37.2)	39 (30.2)	22 (17.1)	15 (11.6)	5 (3.9)	4 (2)
17. Percent of time on diversion	25 (19.4)	36 (27.9)	58 (45.0)	7 (5.4)	3 (2.3)	3 (1)
18. Total ED capacity	47 (36.4)	36 (27.9)	28 (21.7)	16 (12.4)	2 (1.6)	4 (2)
19. Nurse-to-bed ratio	60 (46.5)	35 (27.1)	16 (12.4)	17 (13.2)	1 (0.8)	4 (2)
20. Time from EP assessment to disposition	46 (35.7)	40 (31.0)	27 (20.9)	12 (9.3)	4 (3.1)	4 (2)
21. Time from lab order to lab result returned	68 (52.7)	34 (26.4)	18 (14.0)	7 (5.4)	2 (1.6)	5 (1)
22. Longest time in ED since registration	37 (28.7)	33 (25.6)	40 (31.0)	18 (14.0)	1 (0.8)	4 (2)
23. Number of ED nurses	58 (45.0)	38 (29.5)	21 (16.3)	12 (9.3)	0 (0)	4 (2)
24. Time from physician order to actual imaging	63 (48.8)	41 (31.8)	14 (10.9)	9 (7.0)	2 (1.6)	4 (1)
25. Number of attending ED physicians	58 (45.0)	31 (24.0)	18 (14.0)	17 (13.2)	5 (3.9)	4 (2)
26. Physician-to-patients ratio	66 (51.2)	34 (26.4)	22 (17.1)	4 (3.1)	3 (2.3)	5 (1)
27. Hours of EP coverage	32 (24.8)	31 (24.0)	37 (28.7)	21 (16.3)	32 (24.8)	3 (2)
28. Number of visits during daytime, evening, and overnight	49 (38.0)	43 (33.3)	27 (20.9)	6 (4.7)	4 (3.1)	4 (2)
29. Seasonality of staffing in the ED	38 (29.5)	37 (28.7)	41 (31.8)	11 (8.5)	2 (1.6)	4 (2)
30. Working hours	26 (20.2)	26 (20.2)	41 (31.8)	25 (19.4)	11 (8.5)	3 (2)
31. Time for sub-specialty consultation (orthopedics, cardiology, etc.)	59 (45.7)	46 (35.7)	15 (11.6)	7 (5.4)	2 (1.6)	4 (1)
32. Time between decision to disposition (admission versus discharge) and effective discharge	64 (49.6)	39 (30.2)	14 (10.9)	10 (7.8)	2 (1.6)	4 (1)
33. Ed boarding time	36 (27.9)	35 (27.1)	47 (36.4)	9 (7.0)	2 (1.6)	4 (2)
34. Ability of ambulance to offload	30 (23.3)	25 (19.4)	49 (38.0)	20 (15.5)	5 (3.9)	3 (1)

ED: Emergency department, EP: Emergency physician, IQR: interquartile range

### Second Delphi round

The second Delphi round included the 10 indicators that reached consensus in Round 1. A total of 137 participants completed the second round, with a median (IQR) age of 28 (6). As in Round 1, most participants were male (61.3%) and ER physicians (63.5%). Full demographic details are shown in Table 3.

**Table 3:** Shows demographics of emergency workers included in the second Delphi round (*n* = 137)

Variables		Count	Percent
Age	Mean (SD)	30.55 (7.479)	
	Median (IQR)	28.00 (6)	
Gender	Male	84	61.3
	Female	53	38.7
Position	ER Physicians	87	63.5
	ER Nurse	50	36.5

Table 4 displays the frequency of Likert-scale responses and overall scores for each of the top 10 indicators. Similar to Round 1, the percentage of the ED occupied by inpatients (62%) and the total number of ED patients (56.9%) remained the highest-rated indicators. The number of attending ED physicians received the lowest agreement (34.3%).

**Table 4:** Top 10 ED prolonged LoS indicators: consensus among ED workers

Which elements do you think are most associated with an increase in the length of stay in the emergency department?	Strongly Agree (5)	Agree (4)	Neutral (3)	Disagree (2)	Strongly disagree (1)	Overall score Median (IQR)
1. Total number of ED patients	78 (56.9)	35 (25.5)	14 (10.2)	5 (3.9)	5 (3.9)	5 (1)
2. Overall bed occupancy	60 (43.8)	43 (31.4)	22 (16.1)	7 (5.1)	5 (3.9)	4 (2)
3. Average time in ED for admitted patients since admission	78 (59.9)	33 (24.1)	19 (13.9)	2 (1.5)	5 (3.9)	5 (1)
4. Percentage of ED occupied by inpatients	85 (62.0)	31 (22.6)	16 (11.7)	2 (1.5)	3 (2.2)	5 (1)
5. Time from consultation to disposition decision	62 (45.3)	31 (22.6)	29 (21.2)	12 (8.8)	3 (2.2)	4 (2)
6. Time between decision to disposition (admission versus discharge) and effective discharge	58 (42.3)	36 (26.3)	29 (21.2)	9 (6.6)	5 (3.9)	4 (2)
7. Number of patients boarding in the ED	70 (51.1)	41 (29.9)	21 (15.3)	4 (2.9)	1 (0.7)	5 (1)
8. Time from physician order to actual imaging	53 (38.7)	34 (24.8)	28 (20.4)	15 (10.9)	7 (5.1)	4 (2)
9. Time from lab order to lab result returned	51 (37.2)	31 (22.6)	33 (24.1)	12 (8.8)	10 (7.3)	4 (2)
10. Number of attending ED physicians	47 (34.3)	39 (28.5)	23 (16.8)	19 (13.9)	9 (6.6)	4 (2)

**Fig. 2:**
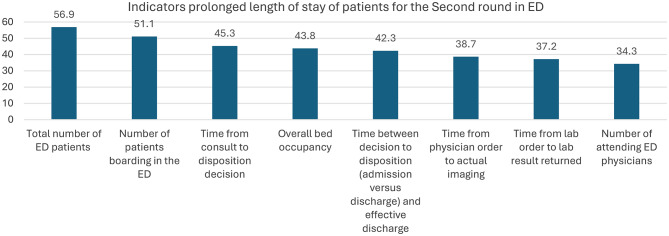
Shows ED workers’ ratings for the top 10 indicators of prolonged LoS in the ED

ED Workers rated the commonly agreed-upon indicators of prolonged LoS for ED patients. The total number of patients was rated highest, and the number of attending physicians was rated lowest (Fig. 2).

By assessing the impact of ED worker demographics on the rating of prolonged LoS indicators (Table 5), several significant differences were observed. Female participants rated the percentage of the ED occupied by inpatients (*p* = 0.011), the time between the decision to disposition and effective discharge (*p* = 0.028), and the number of boarding patients in the ED (*p* = 0.030) as stronger indicators of prolonged LoS.

Additionally, ED nurses rated the average time in the ED for admitted patients since admission (*p* = 0.001), the time from consultation to disposition decision (*p* = 0.014), the time between decision to disposition (*p* = 0.001), and the number of attending ED physicians (*p* = 0.007) as higher indicators of prolonged LoS compared with physicians.

**Table 5:** Impact of demographics of ED workers on ED prolonged LoS indicators’ rating

Variables		Gender		*p* value	Position		*p* value
		Male	Female		Physician (*n* = 87)	Nurse (*n* = 50)	
1. Total number of ED patients	Mean (SD)	4.23 (1.123)	4.38 (0.882)	0.735	4.28 (1.096)	4.30 (0.931)	0.835
	Median (IQR)	5 (1)	5 (1)		5 (1)	5 (1)	
3. Overall bed occupancy	Mean (SD)	3.98 (1.018)	4.21 (1.133)	0.061	3.98 (1.023)	4.22 (1.130)	0.056
	Median (IQR)	4 (2)	5 (1)		4 (2)	5 (1)	
5. Average time in ED for admitted patients since admission	Mean (SD)	4.19 (1.092)	4.45 (0.845)	0.146	4.09 (1.106)	4.64 (0.693)	0.001
	Median (IQR)	5 (1)	5 (1)		4 (1)	5 (0)	
7. Percentage of ED occupied by inpatients	Mean (SD)	4.26 (0.995)	4.64 (0.710)	0.011	4.29 (1.033)	4.62 (0.602)	0.119
	Median (IQR)	5 (1)	5 (1)		5 (1)	5 (1)	
9. Time from consultation to disposition decision	Mean (SD)	3.98 (1.130)	4.04 (1.073)	0.818	3.84 (1.119)	4.28 (1.031)	0.014
	Median (IQR)	4 (2)	4 (2)		4 (2)	5 (1)	
11. Time between decision to disposition	Mean (SD)	3.82 (1.121)	4.21 (1.063)	0.028	3.77 (1.097)	4.32 (1.058)	0.001
	Median (IQR)	4 (2)	5 (1)		4 (2)	5 (1)	
13. Number of patients boarding in the ED	Mean (SD)	4.14 (0.946)	4.49 (0.724)	0.030	4.20 (0.900)	4.42 (0.835)	0.108
	Median (IQR)	4 (1)	5 (1)		4 (1)	5 (1)	
15. Time from physician order to actual imaging	Mean (SD)	3.75 (1.201)	3.91 (1.229)	0.383	3.77 (1.217)	3.88 (1.206)	0.590
	Median (IQR)	4 (2)	4 (2)		4 (2)	4 (2)	
17. Time from lab order to lab result returned	Mean (SD)	3.40 (1.272)	3.83 (1.221)	0.503	3.71 (1.275)	3.78 (1.217)	0.821
	Median (IQR)	4 (2)	4 (2)		4 (2)	4 (2)	
18. Number of attending ED physicians	Mean (SD)	3.64 (1.229)	3.79 (1.306)	0.371	3.48 (1.293)	4.08 (1.104)	0.007
	Median (IQR)	4 (2)	4 (2)		4 (3)	4 (1)	

ED: Emergency department

## Discussion

This study is the first in Saudi Arabia to identify and rank indicators of prolonged LoS in the ED from the perspective of ED staff, providing insight into systemic challenges that contribute to extended stays. LoS in the ED is a critical metric for evaluating quality of care [11, 12]. In the present study, the Delphi method was used to capture expert consensus and develop a deeper understanding of factors influencing prolonged LoS. The Delphi approach is essential for generating expert feedback and improving alignment on complex subjects [13]. Two Delphi rounds were used to evaluate 34 indicators associated with prolonged LoS.

This study identified and ranked 10 indicators considered significant contributors to prolonged ED stays in Saudi Arabia. Among these, the percentage of inpatients occupying the ED emerged as the most influential indicator. This finding emphasizes that ED crowding is not solely an ED-level problem but is tied to the hospital’s overall capacity and inpatient flow. It highlights systemic challenges related to discharge planning and bed management, indicating that inpatient boarding plays a major role in ED congestion and longer LoS [14, 15]. Other patient-level factors, such as older age, higher triage level, and increased severity of illness, further contribute to prolonged stays by increasing care complexity and resource utilization [14].

Indicators ranked highly in the second Delphi round included the average time in the ED for admitted patients, the number of boarding patients, overall bed occupancy, time from consultation to disposition, time from physician order to imaging, and time from lab order to result return. These indicators reflect delays that frequently occur within the ED [16]. Prolonged consultation-to-disposition intervals compound delays in diagnosis and decision-making, thereby worsening crowding and resource strain. These delays reflect multiple factors, including clinical complexity, administrative constraints, and coordination gaps among staff [16].

Delays within the ED are often interconnected and arise from a combination of clinical needs and operational inefficiencies. For example, delays in disposition may result from high patient acuity, workflow interruptions, limited staff availability, or bottlenecks in inpatient admissions. This study found that the number of physicians in the ED was perceived as an indicator of overcrowding and prolonged LoS. However, some studies suggest that physician count alone is not a direct indicator of overcrowding but may instead reflect underlying workflow or resource-allocation challenges [17, 18]. This variation underscores the importance of analyzing how indicators interact. In Saudi EDs, staffing structures often align with accreditation requirements and peak demand periods. This means that simply increasing the number of attending physicians does not guarantee improved flow; factors such as workload distribution, task design, and skill mix may have a greater impact.

International studies support the indicators identified in this research. For example, American studies have found that delays in completing physician orders and delays in receiving laboratory results significantly predict prolonged ED LoS [19]. These findings align with the results of the present study. Delays in laboratory turnaround time often force patients to remain in the ED longer, contributing to overcrowding and extended LoS [20]. In Saudi EDs, laboratory and imaging services—especially for likely admitted patients—may follow inpatient prioritization or centralized scheduling processes, which can further prolong diagnostic timelines during peak hours. Strategies such as point-of-care testing, rapid imaging pathways, and prioritization of electronic orders for ED patients could help mitigate these delays.

Bed occupancy was also found to be strongly associated with prolonged LoS, reflecting inefficiencies in inpatient bed turnover, bed assignment, and discharge processes. High bed occupancy rate can reduce a hospital’s capacity to admit new patients from the ED, increasing ED congestion and lengthening wait times [21]. This strain manifests as extended stays, reduced patient satisfaction, and increased risk of adverse outcomes. Overall bed occupancy and prolonged periods at or above capacity are indicators of access block—situations in which admitted patients cannot be transferred from the ED to inpatient units due to unavailable beds [20]. Access block is recognized as a hospital-wide challenge that affects ED operations [22]. Addressing it requires system-level interventions, such as real-time bed tracking, standardized morning discharge targets, and surge capacity protocols. Incorporating percentage of ED occupied by inpatients and number of ED boarders as live KPIs would allow hospital administrators to respond to leading indicators rather than just lagging outcomes.

Future research should examine how key indicators interact with each other to influence ED flow. Understanding these relationships is essential for developing targeted interventions to improve ED throughput and patient outcomes [14]. Modeling the indicators as an interconnected system—through pathway simulations or time-to-event analyses—could identify threshold effects, such as occupancy levels that trigger increases in consultation delays. Additionally, validating the 8-hour prolonged LoS threshold against local outcomes (e.g., ICU transfers, left-without-being-seen rates) would help refine national benchmarks.

Findings from this study can support the Saudi Ministry of Health’s Ada’a program by integrating real-time monitoring of critical ED indicators, such as inpatient boarding, bed occupancy, and consultation timelines. Tracking these metrics would allow healthcare administrators to implement timely interventions—such as activating discharge lounges or fast-track pathways—when operational thresholds are exceeded. Improvements in lab and imaging turnaround times, streamlined consultation pathways, and optimized bed management could significantly reduce prolonged ED stays and improve patient flow and outcomes.

This study has several limitations. It relies heavily on participants’ subjective responses. Participants were drawn from large urban academic hospitals, which face challenges that may differ from those in rural or non-academic settings. These institutional differences may limit the generalizability of the findings. Future studies should include a more diverse sample from different regions and hospital types, including international contexts, to enhance generalizability. The study also did not explore potential strategies to reduce prolonged LoS.

Additionally, introducing possible bias in panel composition from new participants introduced in Round 2, could disrupt consensus without longitudinal stability checks. Insights from patients and ancillary staff, such as laboratory and radiology personnel, were not included but could provide valuable perspectives on operational barriers affecting LoS. Differences in staffing models, diagnostic access, and transfer logistics between urban and rural hospitals may also influence outcomes and warrant further exploration. Future Delphi panels should consider stratifying participants by institution type and region, tracking changes across rounds, and incorporating broader multidisciplinary input.

## Conclusion

In conclusion, this Delphi study, conducted with experts from Riyadh’s academic hospitals, has prioritized key factors influencing prolonged LoS in the ED, highlighting inpatient boarding and critical diagnostic metrics. To address these issues, we recommend a comprehensive strategy that includes implementing strict policies to limit ED boarding, establishing discharge lounges to expedite bed turnover, enhancing operational benchmarks for diagnostic services, and streamlining consultation processes. Furthermore, the national Ada’a program should integrate these indicators into its performance management structure. These evidence-based recommendations serve as an essential foundation for healthcare leaders across Saudi Arabia to design tailored interventions, with future research focused on evaluating their effectiveness in improving ED efficiency and patient care nationwide.

## Supplementary Information

Below is the link to the electronic supplementary material.

Supplementary Material 1

## Data Availability

The data that support the findings of this study are available from the corresponding author upon reasonable request.

## Abbreviations

IQR: Interquartile Range
LoS: Length of Stay
ED: Emergency Department
