# Supplementary material for: Key indicators contributing to prolonged emergency department stays in Saudi Arabia: a modified Delphi study
Source: BMC Emerg Med. 2025 Dec 15;26:20. doi: 10.1186/s12873-025-01438-y (PMC12821169; doi:10.1186/s12873-025-01438-y)
Supplement: Supplementary file 1 — Supplementary Material 1 [file 12873_2025_1438_MOESM1_ESM.docx]

**Supplementary material**

**First round:**

Which elements do you think are most associated with an increase in the length of stay in the emergency department?

| **Variables with operational definitions** | **Likert Scale** |
| --- | --- |
| **1.Total number of ED patients**  **Total count of individuals registered in the ED within a specific time frame (e.g., per day, week, or month), regardless of acuity or outcome.** | **Strongly agree, agree, neutral, disagree, strongly disagree**  **Strongly agree, agree, neutral, disagree, strongly disagree**  **Strongly agree, agree, neutral, disagree, strongly disagree** |
| **2.Overall bed occupancy**  **Percentage of all available ED beds currently occupied by patients at a given point in time.** |  |
| **3.Number of patients boarding in the ed**  **Number of admitted patients remaining in the ED while awaiting transfer to an inpatient bed.** |  |
| **4.Percentage of ED occupied by inpatients**  **Proportion of ED beds currently occupied by patients who have been admitted but not yet transferred.** |  |
| **5.Time from bed request to bed assignment**  **Average duration between the time a bed is requested for admission and the time a bed is assigned.** |  |
| **6.Number of staffed acute care beds**  **Total number of acute care beds (e.g., ICU, telemetry, medical/surgical) available for patient use and currently staffed.** |  |
| **7.Time from triage to EP**  **Time elapsed between a patient’s triage and their first assessment by an emergency physician.** |  |
| **8.Time from bed ready to transfer to ward**  **Time between when an inpatient bed is marked “ready” and when the patient physically leaves the ED for the ward.** |  |
| **9.Nurse satisfaction**  **A measure of ED nursing staff’s job satisfaction, often assessed through periodic surveys or Likert scale instruments.** |  |
| **10.Time from waiting room to patient care area in ED**  **Time from when a patient enters the waiting room until they are moved to a clinical treatment area.** |  |
| **11.Patients in waiting room**  **Total number of patients currently in the ED waiting room who have not yet been assigned a treatment area.** |  |
| **12.Average time in ED for admitted patient since admission**  **Mean time spent in the ED by patients after admission is ordered, but before they are transferred.** |  |
| **13.Total patients in triage**  **Number of patients currently in the process of triage or waiting to be triaged.** |  |
| **14.Number of patients left without being seen**  **Count of patients who registered at triage but left before receiving a medical screening exam or treatment.** |  |
| **15.Time from consult to disposition decision**  **Time elapsed from when a specialty consultation is requested to when the final disposition decision (admit/discharge) is made.** |  |
| **16.Average and range of patients/hour seen by EP**  **Mean number and variability (range) of patients managed by an emergency physician per hour.** |  |
| **17.Percent of time on diversion**  **Proportion of total operational time that the ED is closed to incoming ambulance traffic due to overcapacity.** |  |
| **18.Total ED capacity**  **Maximum number of patients the ED can safely accommodate, including staffed treatment bays and overflow.** |  |
| **19.Nurse- to-bed ratio**  **Number of nurses on duty divided by the number of available ED beds.** |  |
| **20.Time from EP assessment to disposition**  **Time from first evaluation by an emergency physician to the final disposition decision.** |  |
| **21.Time from lab order to lab result returned**  **Time between when a lab test is ordered and when the result becomes available in the system.** |  |
| **22.Longest time in ED since registration**  **Maximum time any patient has spent in the ED since registering during the reporting period** |  |
| **23.Number of ED nurses**  **Total number of licensed nursing staff assigned to the ED during a defined time period.** |  |
| **24.Time from physician order to actual imaging**  **Time from when an imaging study (e.g., CT, X-ray) is ordered to when the image is taken.** |  |
| **25.Number of attending ED physicians**  **Count of board-certified emergency physicians present and on duty in the ED.** |  |
| **26.Physician-to-patients ratio**  **Number of emergency physicians per patient in the ED at a given time.** |  |
| **27.Hours of EP coverage**  **Total number of hours emergency physicians are scheduled to cover the ED over a specified period (e.g., 24 hours/day).** |  |
| **28.Number of visits during daytime, evening, and overnight**  **Number of patient visits categorized by shifts: Day, Evening, Night.** |  |
| **29.Seasonality of staffing in the ED**  **Variations in ED staffing levels based on predictable seasonal trends (e.g., flu season, holidays).** |  |
| **30.Working hours**  **Scheduled hours of operation for ED clinical and support staff (physicians, nurses, radiology, labs, etc.).** |  |
| **31.Time for sub-specialty consultation (orthopedics, cardiology, etc.)**  **Time between when a sub-specialty consult (e.g., ortho, cardiology) is requested and when the consultant sees the patient.** |  |
| **32.Time between decision to disposition (admission versus discharge) and effective discharge**  **Time between the disposition decision (discharge or admit) and when the patient physically leaves the ED.** |  |
| **33.Ed boarding time**  **Time an admitted patient spends in the ED after the admission decision until transfer to an inpatient bed.** |  |
| **34.Ability of ambulance to offload**  **Measure of how quickly and consistently ambulances can transfer patients to ED staff upon arrival. May be represented as offload delay time or percentage within a target time (e.g., within 15 minutes).** |  |

**Second round:**

Which elements do you think are most associated with an increase in the length of stay in the emergency department?

| **Variables with operational definitions** | **Likert Scale** |
| --- | --- |
| **1.Total number of ED patients**  **Total count of individuals registered in the ED within a specific time frame (e.g., per day, week, or month), regardless of acuity or outcome.** | **Strongly agree, agree, neutral, disagree, strongly disagree** |
| **2.Overall bed occupancy**  **Percentage of all available ED beds currently occupied by patients at a given point in time.** |  |
| **3.Average time in ED for admitted patient since admission**  **Mean time spent in the ED by patients after admission is ordered, but before they are transferred.** |  |
| **4.Percentage of ED occupied by inpatients**  **Proportion of ED beds currently occupied by patients who have been admitted but not yet transferred.** |  |
| **5.Time from consult to disposition decision**  **Time elapsed from when a specialty consultation is requested to when the final disposition decision (admit/discharge) is made.** |  |
| **6.Time between decision to disposition (admission versus discharge) and effective discharge**  **Time between the disposition decision (discharge or admit) and when the patient physically leaves the ED.** |  |
| **7.Number of patients boarding in the ed**  **Number of admitted patients remaining in the ED while awaiting transfer to an inpatient bed.** |  |
| **8.Time from physician order to actual imaging**  **Time from when an imaging study (e.g., CT, X-ray) is ordered to when the image is taken.** |  |
| **9.Time from lab order to lab result returned**  **Time between when a lab test is ordered and when the result becomes available in the system.** |  |
| **10.Number of attending ED physicians**  **Count of board-certified emergency physicians present and on duty in the ED.** |  |
